# Airway inflammatory changes in the lungs of patients with asthma-COPD overlap (ACO): a bronchoscopy endobronchial biopsy study

**DOI:** 10.1186/s12931-023-02527-x

**Published:** 2023-09-12

**Authors:** Surajit Dey, Wenying Lu, Greg Haug, Collin Chia, Josie Larby, Heinrich C. Weber, Archana Vijay Gaikwad, Prem Bhattarai, Affan Mahmood Shahzad, Prabuddha S. Pathinayake, Peter A. B. Wark, Mathew Suji Eapen, Sukhwinder Singh Sohal

**Affiliations:** 1 Respiratory Translational Research Group, Department of Laboratory Medicine, School of Health Sciences, College of Health and Medicine, University of Tasmania, Locked Bag, 1322, Newnham Drive, Launceston, TAS 7248 Australia; 2 Launceston Respiratory and Sleep Centre, Launceston, TAS 7250 Australia; 3 Department of Respiratory Medicine, Launceston General Hospital, Launceston, TAS 7250 Australia; 4 Department of Respiratory Medicine, Tasmanian Health Services (THS), North-West Hospital, Burnie, TAS Australia; 5 Immune Health Program, Hunter Medical Research Institute, University of Newcastle, New Lambton Heights, Australia; 6 Department of Respiratory and Sleep Medicine, John Hunter Hospital, New Lambton Heights, Australia

**Keywords:** ACO, Asthma, COPD, Inflammatory cells, Macrophage, CD + 8 T-cells, Mast cells

## Abstract

**Background:**

Although asthma and chronic obstructive pulmonary disease (COPD) are two distinct chronic airway inflammatory diseases, they often co-exist in a patient and the condition is referred to as asthma-COPD overlap (ACO). Lack of evidence regarding the inflammatory cells in ACO airways has led to their poor prognosis and treatment. The objective of this endobronchial biopsy (EBB) study was to enumerate inflammatory cellular changes in the airway wall of ACO compared with asthma, COPD current smokers (CS) and ex-smokers (ES), normal lung function smokers (NLFS), and non-smoker controls (HC).

**Methods:**

EBB tissues from 74 patients were immunohistochemically stained for macrophages, mast cells, eosinophils, neutrophils, CD8+ T-cells and CD4+ T-cells. The microscopic images of stained tissues were evaluated in the epithelium, reticular basement membrane (RBM) cells/mm RBM length, and lamina propria (LP) cells/mm^2^ up to a depth of 120 µM using the image analysis software Image-Pro Plus 7.0. The observer was blinded to the images and disease diagnosis. Statistical analysis was performed using GraphPad Prism v9.

**Results:**

The tissue macrophages in ACO were substantially higher in the epithelium and RBM than in HC (*P* < 0.001 for both), COPD-ES (*P* < 0.001 for both), and -CS (*P* < 0.05 and < 0.0001, respectively). The ACO LP macrophages were significantly higher in number than COPD-CS (*P* < 0.05). The mast cell numbers in ACO were lower than in NLFS (*P* < 0.05) in the epithelium, lower than COPD (*P* < 0.05) and NLFS (*P* < 0.001) in RBM; and lower than  HC (*P* < 0.05) in LP. We noted lower eosinophils in ACO LP than HC (*P* < 0.05) and the lowest neutrophils in both ACO and asthma. Furthermore, CD8+ T-cell numbers increased in the ACO RBM than HC (*P* < 0.05), COPD-ES (*P* < 0.05), and NLFS (*P* < 0.01); however, they were similar in number in epithelium and LP across groups. CD4+ T-cells remained lower in number across all regions and groups.

**Conclusion:**

These results suggest that the ACO airway tissue inflammatory cellular profile differed from the contributing diseases of asthma and COPD with a predominance of macrophages.

## Introduction

Asthma and chronic obstructive pulmonary disease (COPD) are the most prevalent chronic inflammatory and heterogeneous airway diseases. These two diseases can co-exist in a patient, resulting in a clinical phenotype asthma-COPD overlap (ACO) [1–3]. Patients with ACO have been a focus of interest in recent years, because of their poor health-related quality of life, increased comorbidities, and greater healthcare utilization, leading to a greater socioeconomic burden [4].

The overall clinical pictures in asthma and COPD are always influenced by the development of inflammatory processes, i.e., neutrophilic, eosinophilic, or mixed [1–3]. So far, blood, bronchoalveolar lavage (BAL), or lung tissue studies revealed that airway inflammation is the central component of these two obstructive airway diseases [1]. The asthma biopsy studies showed abundant tissue eosinophils, mast cells, and cluster of differentiation (CD)4 + T lymphocytes [1, 5, 6]. Neutrophilia, an increase in macrophages, and CD8+ T-cells are the prominent inflammatory features of COPD tissues [6–9]. Nonetheless, some literature indicated an inconsistent finding on inflammatory cell types in both diseases: asthmatic smokers showed a severe disease with tissue neutrophilia alongside the fixed airflow obstruction [1, 10], whereas eosinophilic inflammation was also reported in COPD patients [11]. Therefore, both diseases are vastly heterogeneous, with multiple and superimposed inflammatory and clinical phenotypes such as ACO.

Despite exhaustive research on asthma and COPD, identification of an inflammatory biomarker remains elusive for ACO. Although the blood and sputum studies provided specific information on inflammatory biomarkers [1], the numbers of original research examining the inflammatory cellular pattern right at the site of inflammation i.e., the airway tissue of ACO patients, is rare. A recent study [12] reported a similar tissue lymphocyte and eosinophilic infiltration, number of granulocytes in the stroma and the epithelium of COPD patients with and without asthma characteristics; however, the study lacked a comparison with normal subjects. Nevertheless, our recent data on airway tissue cellularity of ACO patients suggested a trend of hypercellular LP compared to COPD-CS and normal subjects, and hypocellular LP compared to asthma [13]. Consequently, we further questioned whether any cell types predominate the ACO airway tissues.

Therefore, in this cross-sectional exploratory study, we performed an in-depth analysis of inflammatory cells, including macrophages, neutrophils, eosinophils, mast cells, CD8+ , and CD4+ T lymphocytes in the large airway EBB tissues of ACO, especially in the epithelium, RBM, and LP area, and compared them with asthma, COPD-ES and COPD-CS, HC, and NLFS.

## Methods

This study used participants’ data from two Cohorts, viz, Cohort A and Cohort B. Cohort B is a historical data cohort from our laboratory published by Eapen MS et al. (Table 1) [8].

**Table 1:** Participant demography and lung function data

	Cohort A^1,2^						Cohort B^3^			
Groups	HC	ACO	Asthma	COPD-ES	COPD-CS	NLFS	HC	COPD- ES	COPD-CS	NLFS
Subjects	14	12	14	10	12	12	25	14	13	20
Age (years)	66 (20–76)	70 (52–77)	62 (26–81)	67 (46–78)	65.5 (51–78)	56.5 (41–72)	44 (20–68)	62 (53–69)	61 (46–78)	50 (30–66)
Smoking History (pack-year)	0	22.5 (15–103)	0	36 (22–105)	34.8 (10–114)	31.5 (20–75)	0	51 (18–150)	45 (18–78)	32 (10–57)
ICS treatment (n)	N/A	7	6	N/A	N/A	N/A	N/A			
ICS dose	N/A	800 mcg/day	750 mcg/day	N/A	N/A	N/A	N/A			
GINA Diagnosis Mild persistent/ Moderate/Severe (n)		0/0/4	5/1/8	N/A	N/A	N/A	N/A			
Gold Diagnosis Stage I &II/Stage III (n)		7/1	N/A	10/0	12/0	N/A	N/Av			
%FEV_1_	90 (78–114)	58 (35–96)	81.5 (48–108)	84.5 (54–113)	69 (49–92)	94 (79–113)	113 (86–140)	83 (54–104)	83 (66–102)	99 (78–125)
%FEV_1_/FVC	80 (71–84)	65.5 (31–84)	74 (52–90)	63.2 (55–69)	63.6 (50–75.3)	78 (70–85)	82 (71–88)	57 (38–68)	59 ± 7.6	80.6 (77–86)
BDR (mL)	–	201 (200–800)	–	–	–	–	–	–	–	–
BDR%	–	19 (12–38)	–	–	–	–	–	–	–	–
Atopy (n)		3	7	–	–	–	–	–	–	–
BAL macrophages (%)	–	27.5 (4.3–68.5)	27.9 (2.5–76.3)	–	–	–	–	–	–	–
BAL eosinophils (%)	–	3.8 (71.8–0)	1.9 (0–42.7)	–	–	–	–	–	–	–
BAL neutrophils (%)	–	40.6 (13–92.5)	41.6 (6.7–93.5)	–	–	–	–	–	–	–

Data expressed as median and range

*ACO*= a﻿sthma-COPD overlap, *BDR*= bronchodilator reversibility, *COPD*= chronic obstructive pulmonary disease, *COPD-CS*= COPD current smokers, *COPD-ES*= COPD ex-smokers, *FEV1*= forced expiratory volume in 1 s, *FVC*= forced vital capacity, *GINA*= The Global Initiative for Asthma, *GOLD*= The Global Initiative for Chronic Obstructive Lung Disease, *HC*= healthy control, *n*= number of subjects/patients, *N/A*= not applicable, *N/Av*= not available, *NLFS*= normal lung function smokers, *ICS*= inhaled corticosteroids

^1^All groups of Cohort A were used for comparing mast cells and eosinophils

^2^The ANOVA results of age comparison between groups were not statistically significant. ANOVA results of %FEV_1_/FVC between groups showed significant *P* values between HC and all pathological groups, and between NLFS and all pathological groups

^3^All groups from Cohort B, and ACO and asthma groups of Cohort A were used for comparison of macrophage, neutrophils, CD8+ T-cells and CD4+ T-cells

### Participant demographics

For Cohort A, 74 large airway EBB samples collected from participants (12 ACO, 14 asthmatics, 22 COPD, 14 HC, and 12 NLFS), were obtained from the Tasmanian Respiratory Tissue Bank and Newcastle Biobank (Tasmanian Health and Medical Human Research Ethics Committee, ethics ID: H0013051; the Hunter New England Human Research Ethics Committee reference no: 05/08/10/3.09). A description of tissue collection from the research volunteers was published in our earlier report [13]. The entire Cohort A was used for the comparison of mast cells and eosinophils. In addition, the ACO and asthma EBB samples from Cohort A were clubbed with the HC, COPD-ES, COPD-CS, and NLFS groups of Cohort B (historical data) to compare the macrophages, neutrophils, CD8+ T-cells and CD4+ T-cells.

ACO patients were defined with a combination of asthma and COPD definitions, i.e., patients with a history of asthma, allergies, or atopy in combination with a postbronchodilator FEV1 < 80% of predicted value and FEV_1_/FVC < 70% plus an increase in postbronchodilator FEV_1_ or FVC ≥ 200 mL and ≥ 12%. In Cohort A, 7 participants with ACO were classified as GOLD stage I and II, 1 participant with GOLD stage III COPD, and four were classified as having severe asthma. Most participants with ACO were ex-smokers. A summary of the participant demographics is presented in Table 1.

### Immunohistochemical staining

Formalin-fixed, paraffin-embedded biopsy tissue blocks were sectioned at 3 µm thickness. Following epitope retrieval, immunostaining with primary antibodies monoclonal anti-human CD68 (1:500 dilution, clone KP1, M0814, Dako, Victoria, Australia), monoclonal mouse anti-human neutrophil elastase (1:200 dilution, clone NP57, M0752, Dako, Victoria, Australia), recombinant anti-ribonuclease 3 eosinophil cationic protein (1:8000 dilution, ab207429, Abcam, Victoria, Australia), mast cell tryptase (1:400 dilution, clone AA1, M7052, Dako, Victoria, Australia), mouse anti-human CD8 (1:30 dilution, clone 4B11, NCL-CD8-4B11-L-CE, Leica Biosystems, Victoria, Australia) and mouse anti-human CD4 (1:30 dilution, clone 4B12, M7310, Dako, Victoria, Australia) was performed at room temperature. Polymer enzyme backbone conjugated secondary antibody (Dako EnVision, K5007) was used after the primary antibodies and visualized using diaminobenzidine chromogen (Dako EnVision, K7005). Harris haematoxylin was used to stain the nuclear component. Optimization of the manufacturer-specific immunohistochemical method was undertaken before batch immunostaining with specific inflammatory markers.

### Quantification of immuno-stained endobronchial biopsy tissue

**Fig. 1:**
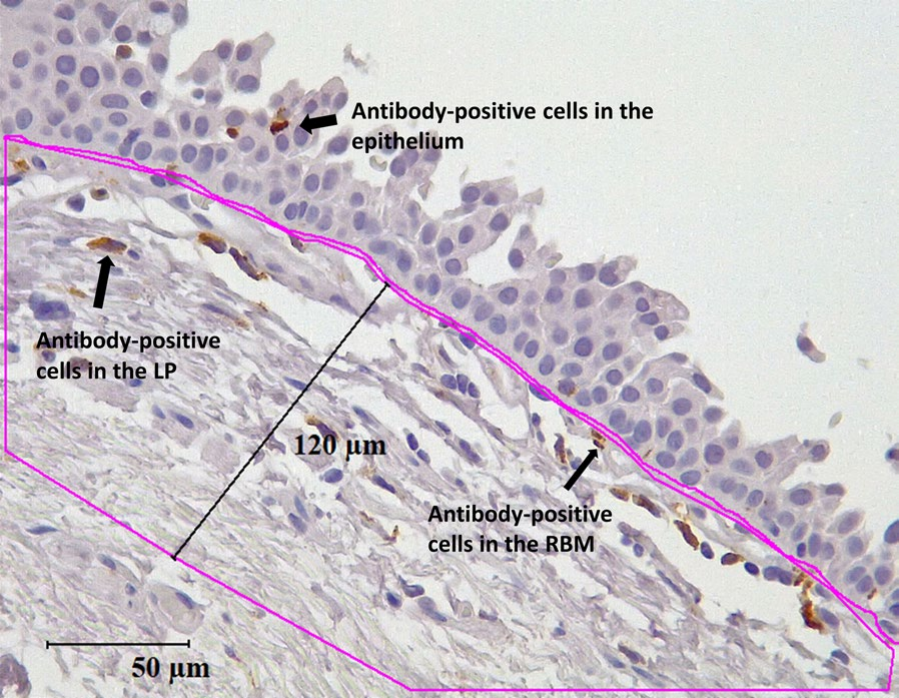
Representation of antibody-positive cells that were counted in the epithelial, reticular basement membrane (RBM), and lamina propria (LP) areas

Computer-assisted image analysis was performed using a Leica DM 500 microscope (Leica Microsystems, Germany) and a Leica ICC50W camera. Before capturing the images, the slides were blinded to the diagnosis and subjects. Tissues with visible epithelium, RBM, and LP were elected for image analysis. In brief, images of the entire tissue were captured at 40X brightfield, and overlapping was strictly avoided. Five out of the total images of each slide were randomly selected for cell counts. The quantification of positive marker cells (brown) in the epithelial, RBM, and LP up to 120 µm deep inside the tissue was performed using the image analysis software Image-Pro Plus 7.0 (Fig. 1). The RBM cells that were within the two lines of basal surface of epithelium, and another trace line at outer limit of lamina reticularis of RBM including cells moving away from it toward the LP, were included in RBM cell counting. The quantified cells in the epithelium and RBM were presented per mm of RBM length, and the cells in the LP were presented per mm^2^ of LP area.

### Statistical analysis

Data distribution was evaluated using D'Agostino & Pearson test, and intra- and inter-group variances were analyzed using one way ANOVA and Kruskal–Wallis (nonparametric) with multiple comparisons using unadjusted Dunn's test. Results are reported as median and range unless otherwise mentioned. Based on the ANOVA result, univariate Spearman *r* was used for correlation analysis. Furthermore, the effect of inhaled corticosteroids (ICS) on inflammatory cells was explored using a nonparametric test (Mann–Whitney test). A *P*-value of < 0.05 was considered significant. All analyses were done using GraphPad Prism v9 (San Diego, CA, USA).

## Results

### Macrophages

**Fig. 2:**
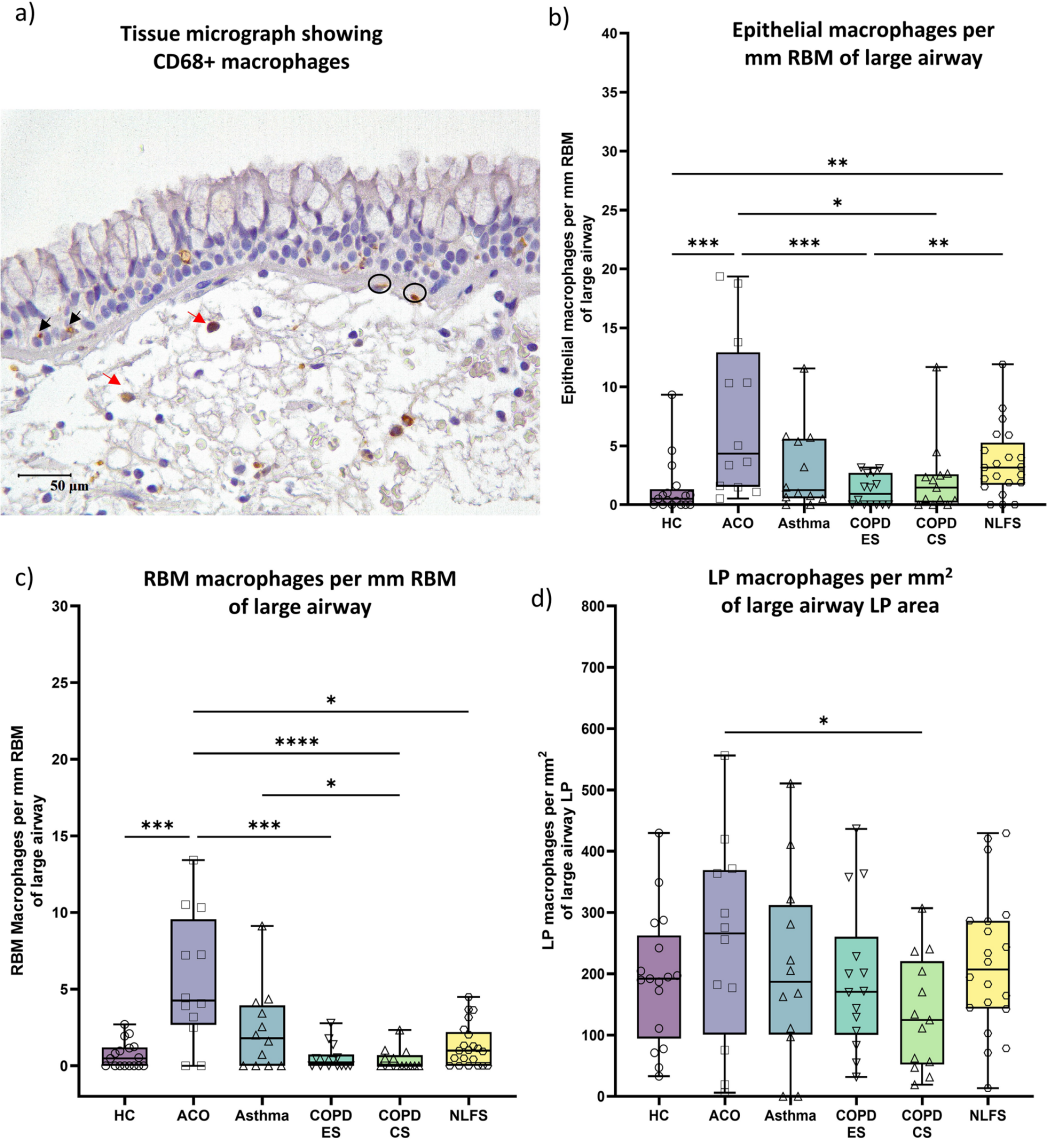
Representative tissue micrograph (**a**) for CD68+ cells in the epithelium (black arrow), reticular basement membrane (RBM) (circled), and lamina propria (LP) (red arrow). Box plots showing CD68 + macrophages in the large airway epithelium (**b**), RBM (**c**), and LP (**d**) of healthy control (HC), asthma COPD overlap (ACO), asthma, chronic obstructive pulmonary disease (COPD) ex–smokers (ES) and current smokers (CS), and normal lung function smokers (NLFS). The horizontal line inside each box represents the median; the top and bottom of each box represent the upper and lower quartiles, respectively; and the whiskers represent extreme values. ANOVA *P-*value representation * < 0.05, ** < 0.01, *** < 0.001, **** < 0.0001. Insignificant *P* values are not shown in the plot

The CD68+ macrophages (Fig. 2a) were highest in ACO epithelium, RBM, and LP area among the groups evaluated (Fig. 2b–d). As compared to HC, we noted a significantly high epithelial (*P* < 0.001) and RBM (*P* < 0.001), and an insignificantly high LP macrophage count in ACO. In addition, ACO macrophages were substantially higher in number in all three regions (epithelium *P* < 0.05, RBM *P* < 0.0001, and LP *P* < 0.05) compared to COPD-CS. Similarly, the ACO macrophages were notably higher in number in the epithelium and RBM regions and insignificantly higher in number in the LP than the COPD-ES. Although we found more macrophages in the epithelium, RBM, and LP regions of ACO compared to asthma, the differences were statistically insignificant. In addition, there were notably higher macrophage numbers in the RBM of asthma than in COPD-ES (*P* < 0.05).

### Neutrophils

**Fig. 3:**
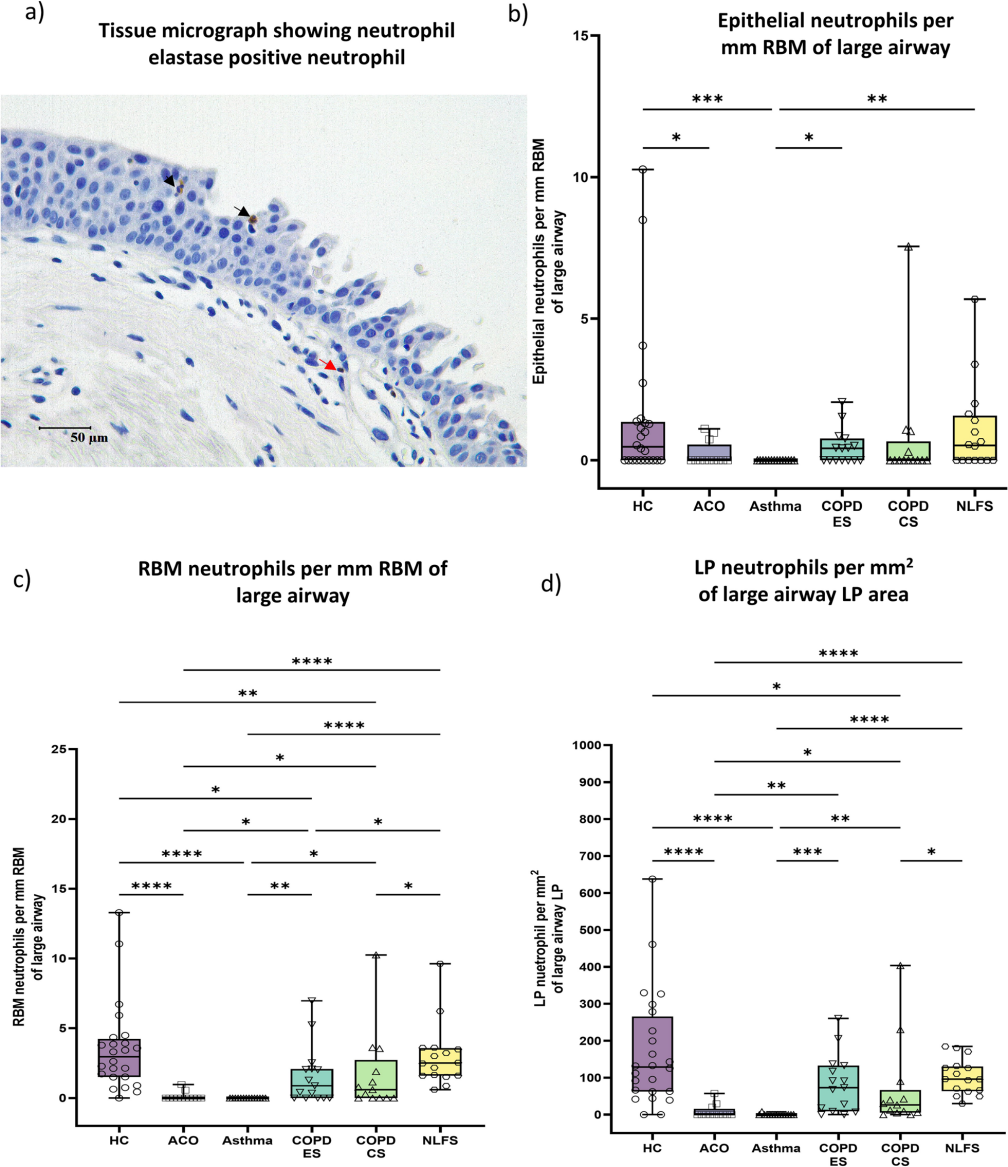
Representative tissue micrograph (**a**) for neutrophil elastase positive cells in the epithelium (black arrow) and lamina propria (LP) (red arrow). Box plots showing neutrophils in the large airway epithelium (**b**), reticular basement membrane (RBM) (**c**), and LP (**d**) of healthy control (HC), asthma COPD overlap (ACO), asthma, chronic obstructive pulmonary disease (COPD) ex–smokers (ES) and current smokers (CS), and normal lung function smokers (NLFS). The horizontal line inside each box represents the median; the top and bottom of each box represent the upper and lower quartiles, respectively; and the whiskers represent extreme values. ANOVA *P-*value representation * < 0.05, ** < 0.01, *** < 0.001, **** < 0.0001. Insignificant *P* values are not shown in the plot

In general, we noted very few neutrophils (Fig. 3a) in ACO and asthma groups. In fact, we noted these neutrophil counts are in only 3 ACO patients. Overall, compared to HC, the number of neutrophils was significantly low in the ACO epithelium (*P* < 0.05), RBM (*P* < 0.0001), and LP (*P* < 0.0001) regions (Fig. 3b–d). Similarly, ACO neutrophils in RBM and LP remained substantially lower than the NLFS (*P* < 0.0001 for both). Compared to COPD ES and CS, the neutrophil counts were also significantly low in ACO RBM (*P* < 0.05) and LP (*P* < 0.05 and *P* < 0.01, respectively). In addition, compared to HC and NLFS, the number of neutrophils was substantially lower in the COPD-CS RBM (*P* < 0.01, < 0.05, respectively) and LP (*P* < 0.05), and in the RBM of COPD-ES (*P* < 0.05) [8].

### Eosinophils

**Fig. 4:**
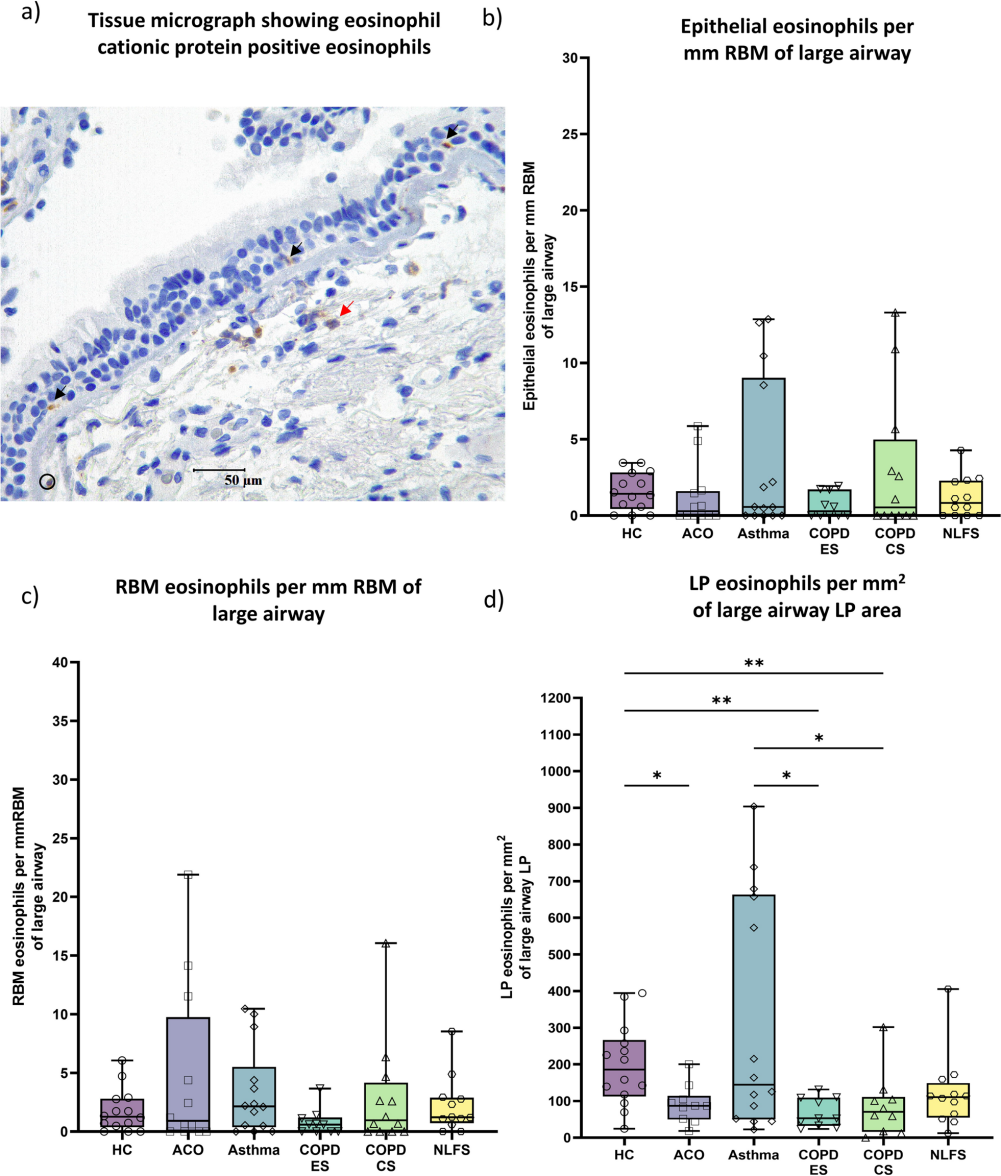
Representative tissue micrograph (**a**) for eosinophil cationic protein positive cells in the epithelium (black arrow) and lamina propria (LP) (red arrow). Box plots showing eosinophils in the large airway epithelium (**b**), reticular basement membrane (RBM) (**c**), and LP (**d**) of healthy control (HC), asthma COPD overlap (ACO), asthma, chronic obstructive pulmonary disease (COPD) ex–smokers (ES) and current smokers (CS), and normal lung function smokers (NLFS). The horizontal line inside each box represents the median; the top and bottom of each box represent the upper and lower quartiles, respectively; and the whiskers represent extreme values. ANOVA *P-*value representation * < 0.05, ** < 0.01, *** < 0.001, **** < 0.0001. Insignificant *P* values are not shown in the plot

While eosinophilic cell (Fig. 4a) density in the epithelial and RBM regions was similar across the groups, in contrast, when compared to HC, lower eosinophil numbers were  found in the LP of ACO, and COPD (Fig. 4b–d). In the ACO LP, the number of eosinophils was significantly lower (*P* < 0.05) than in HC. Although the eosinophil numbers in the ACO LP were comparable to those in the COPD-ES or CS groups, the ACO eosinophils tended to be lower in number, which was statistically insignificant than asthma. In addition, we noted noticeably low LP eosinophils in COPD-ES and CS groups compared to HC (*P* <0.01) and asthma (*P* <0.05).

### Mast cells

**Fig. 5:**
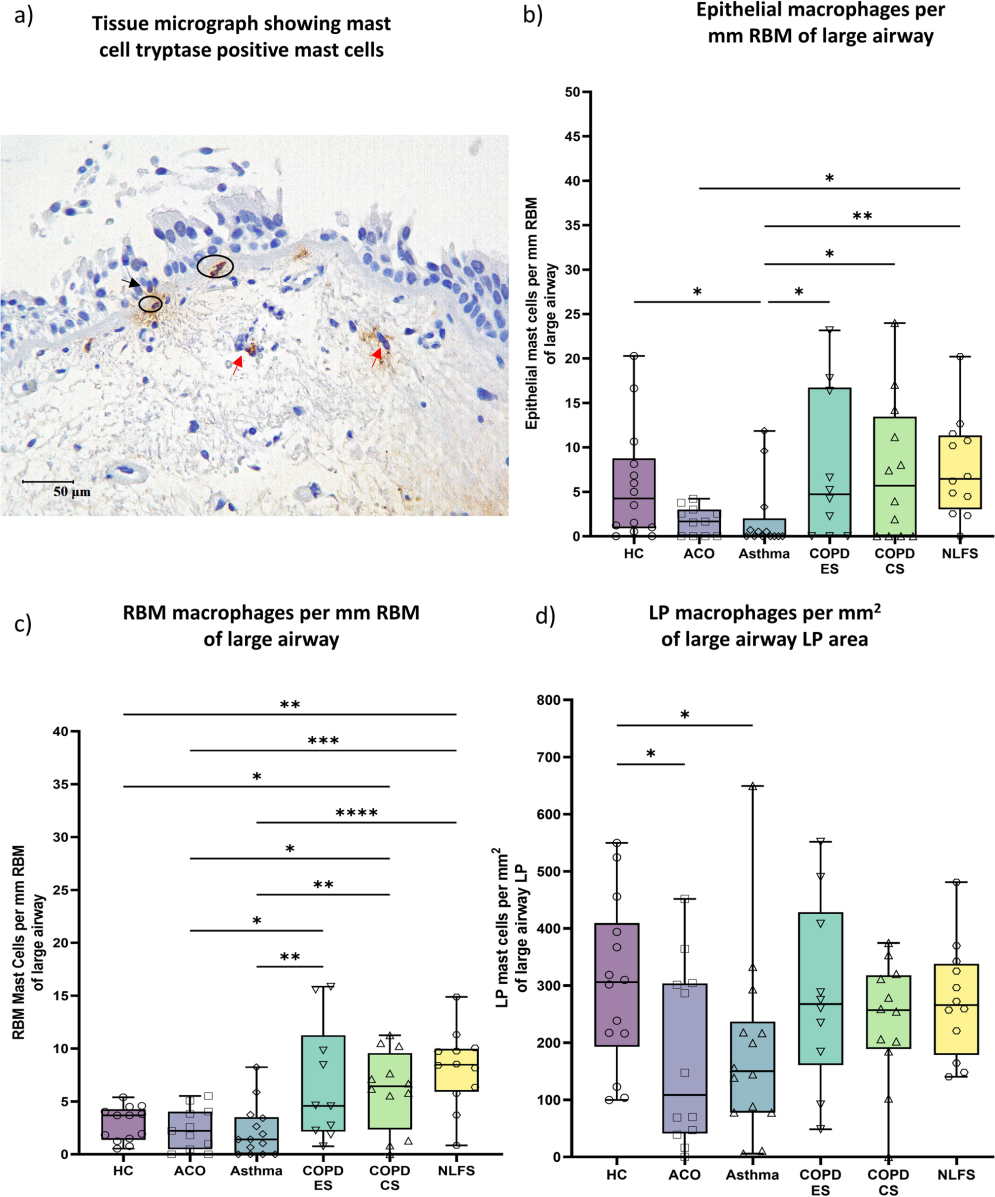
Representative tissue micrograph (**a**) for mast cell tryptase positive cells in the epithelium (black arrow), reticular basement membrane (RBM) (circled), and in lamina propria (LP) (red arrow). Box plots showing mast cells in the large airway epithelium (**b**), reticular RBM (**c**), and LP (**d**) of healthy control (HC), asthma COPD overlap (ACO), asthma, chronic obstructive pulmonary disease (COPD) ex–smokers (ES) and current smokers (CS), and normal lung function smokers (NLFS). The horizontal line inside each box represents the median; the top and bottom of each box represent the upper and lower quartiles, respectively; and the whiskers represent extreme values. ANOVA *P-*value representation * < 0.05, ** < 0.01, *** < 0.001, **** < 0.0001. Insignificant *P* values are not shown in the plot

Mast cell (Fig. 5a) numbers appeared to be lower in ACO epithelium and RBM than in the HC and significantly lower (*P* < 0.05) in the LP region than in HC (Fig. 5b–d). Further, mast cell count in ACO epithelium was substantially lower (*P* < 0.05) compared to the NLFS and tended to be lower than the COPD groups although differences were statistically insignificant. In the RBM, mast cells were also lower in ACO than the COPD groups (*P* < 0.05) and NLFS (*P* < 0.001); however, it was comparable with asthma. In the LP region, mast cell numbers were lowest in ACO among the groups and the differences were statistically insignificant when compared against asthma, COPD-ES or CS, and NLFS (Fig. 5d). Furthermore, we noted significantly lower mast cells in the epithelium and LP of asthma compared to HC (*P* < 0.05). Mast cells were also significantly lower in asthma epithelium and RBM as compared to COPD-ES (*P* < 0.05, < 0.05, respectively) and CS (*P* < 0.05, < 0.05, respectively). Cell density in COPD-ES and CS were similar in all three regions evaluated.

### CD8 + T-cells

**Fig. 6:**
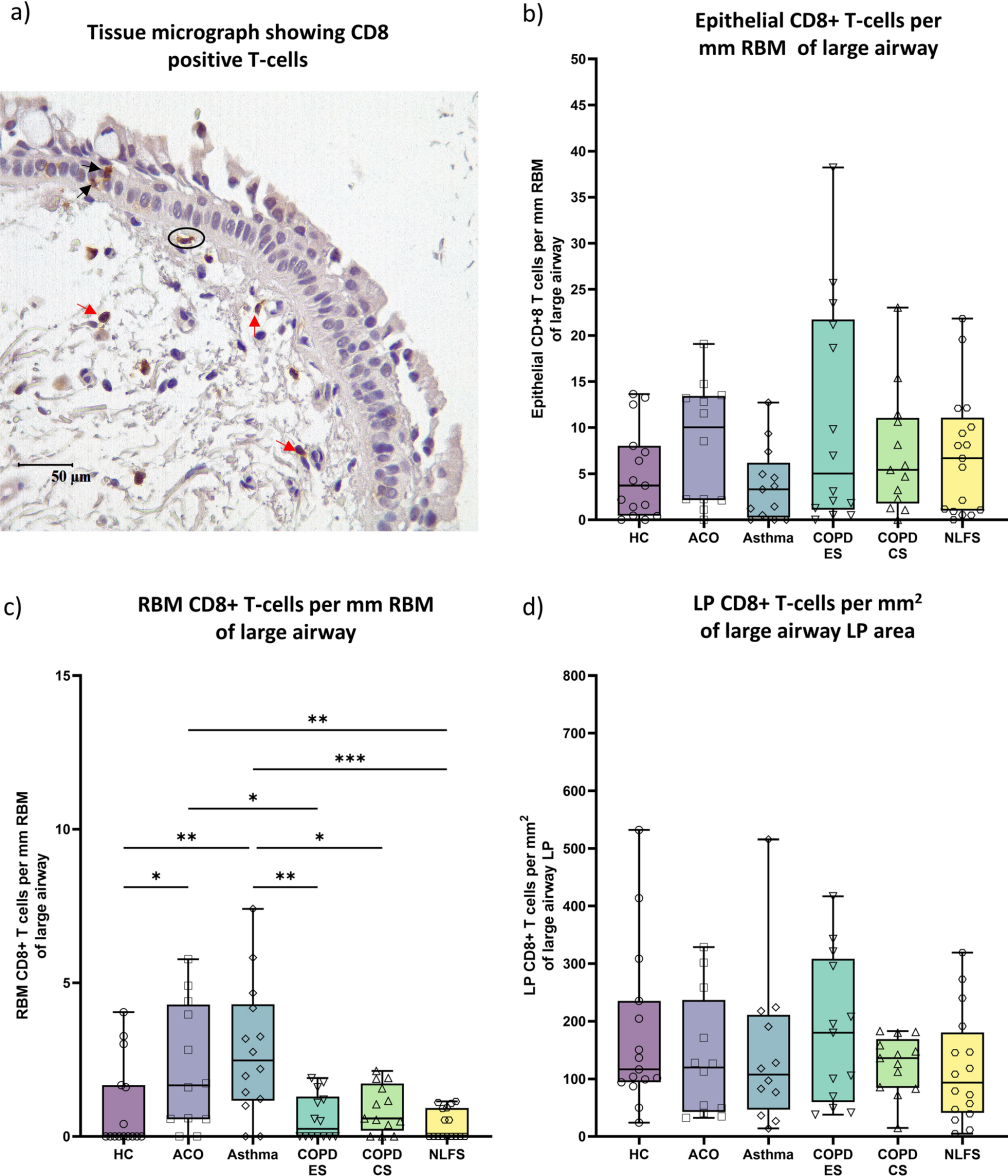
Representative tissue micrograph (**a**) for CD8+ T-cells in the epithelium (black arrow), reticular basement membrane (RBM) (circled), and lamina propria (LP) (red arrow). Box plots showing CD8 + T cells in the large airway epithelium (**b**), RBM (**c**), and LP (**d**) of healthy control (HC), asthma COPD overlap (ACO), asthma, chronic obstructive pulmonary disease (COPD) ex–smokers (ES) and current smokers (CS), and normal lung function smokers (NLFS). The horizontal line inside each box represents the median; the top and bottom of each box represent the upper and lower quartiles, respectively; and the whiskers represent extreme values. ANOVA *P-*value representation * < 0.05, ** < 0.01, *** < 0.001, **** < 0.0001. Insignificant *P* values are not shown in the plot

The CD8+ T-cells (Fig. 6a) dominated the epithelial region of the ACO group, however, compared to other groups, the differences were not statistically significant (Fig. 6b). The RBM CD8 + T cell numbers in ACO was significantly higher than the HC (*P* < 0.05), COPD-ES (*P* < 0.05), and NLFS (*p* < 0.01) and tended to be lower than the asthmatic, but without statistical significance (Fig. 6c). Furthermore, we noticed the highest number of RBM CD8 + T-cells in the asthma group, which was substantially different from the HC (*P* < 0.01), COPD-ES (*P* < 0.01), and CS (*p* < 0.05), and NLFS (*P* < 0.001). In the LP region, CD8 + T-cells were similar in numbers in HC, ACO, asthma, COPD-CS, and NLFS groups (Fig. 6d). The number of CD8 + T-cells was highest in the COPD-ES group and tended to be higher than the HC, although the difference was statistically insignificant [8].

### CD4 + T-cells

**Fig. 7:**
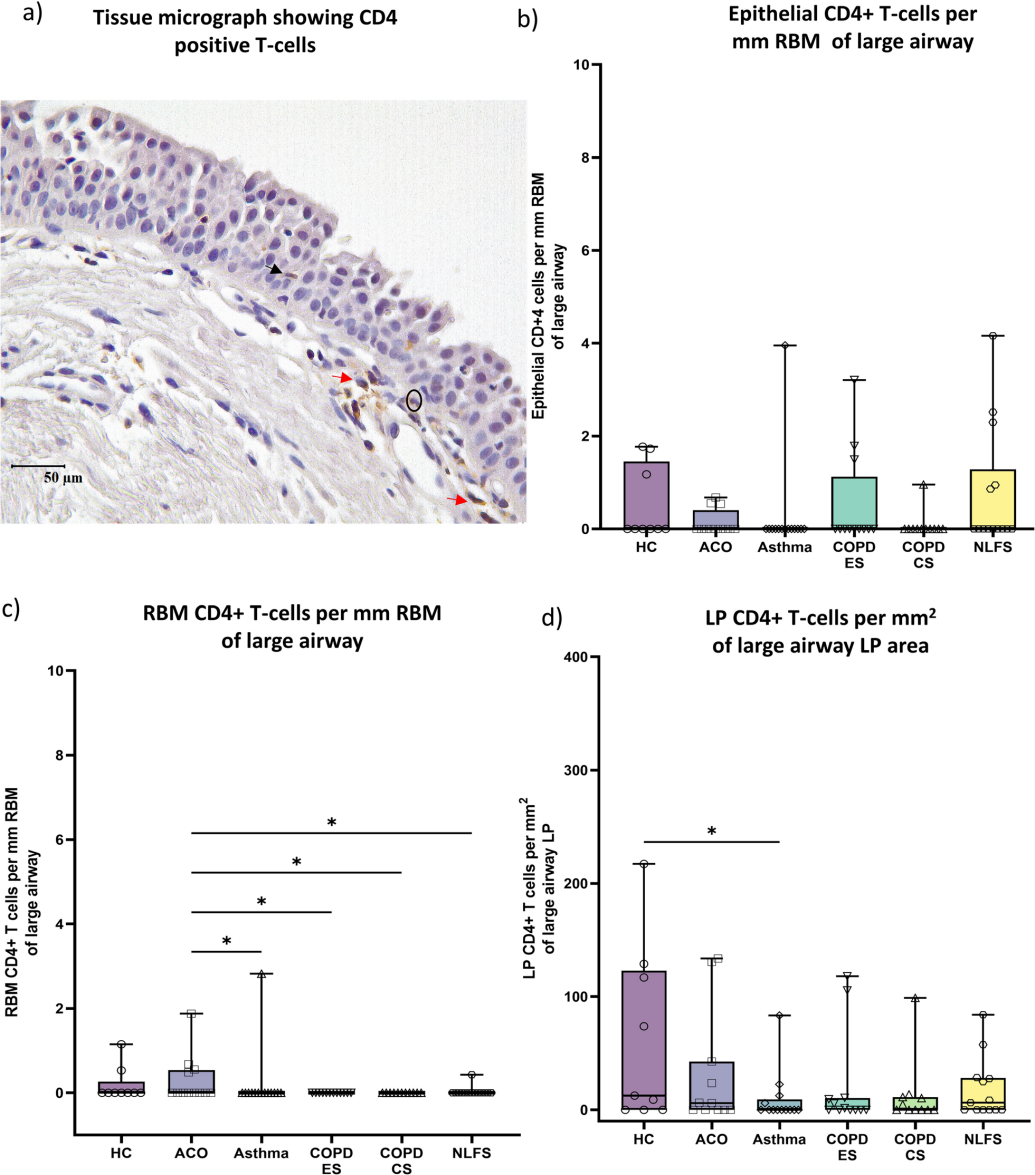
Representative tissue micrograph (**a**) for CD4+ T cells in the epithelium (black arrow), reticular basement membrane (RBM) (circled), and lamina propria (LP) (red arrow). Box plots showing CD4 + T-cells in the large airway epithelium (**b**), RBM (**c**), and LP (**d**) of healthy control (HC), asthma COPD overlap (ACO), asthma, chronic obstructive pulmonary disease (COPD) ex–smokers (ES) and current smokers (CS), and normal lung function smokers (NLFS). The horizontal line inside each box represents the median; the top and bottom of each box represent the upper and lower quartiles, respectively; and the whiskers represent extreme values. ANOVA *P-*value representation * < 0.05, ** < 0.01, *** < 0.001, **** < 0.0001. Insignificant *P* values are not shown in the plot

We noticed a low number of CD4 + T cells (Fig. 7a), and the cell density was similar in the epithelial, RBM, and LP regions across the groups (Fig. 7b–d). Although the high number of RBM CD4 + T-cells in ACO showed a statistical significance as compared to asthma, COPD-ES (*P* < 0.05), CS (*P* < 0.05), and NLFS (*P* < 0.05), it should be borne in mind that CD4 + T-cells in the RBM were noted only in 4 ACO patients, 1 asthma patients, and in 1 NLFS. Furthermore, we observed a low number of LP CD4 + T-cells in asthmatics compared to HC (*P* < 0.05).

### Exploratory analysis and corelations

**Fig. 8:**
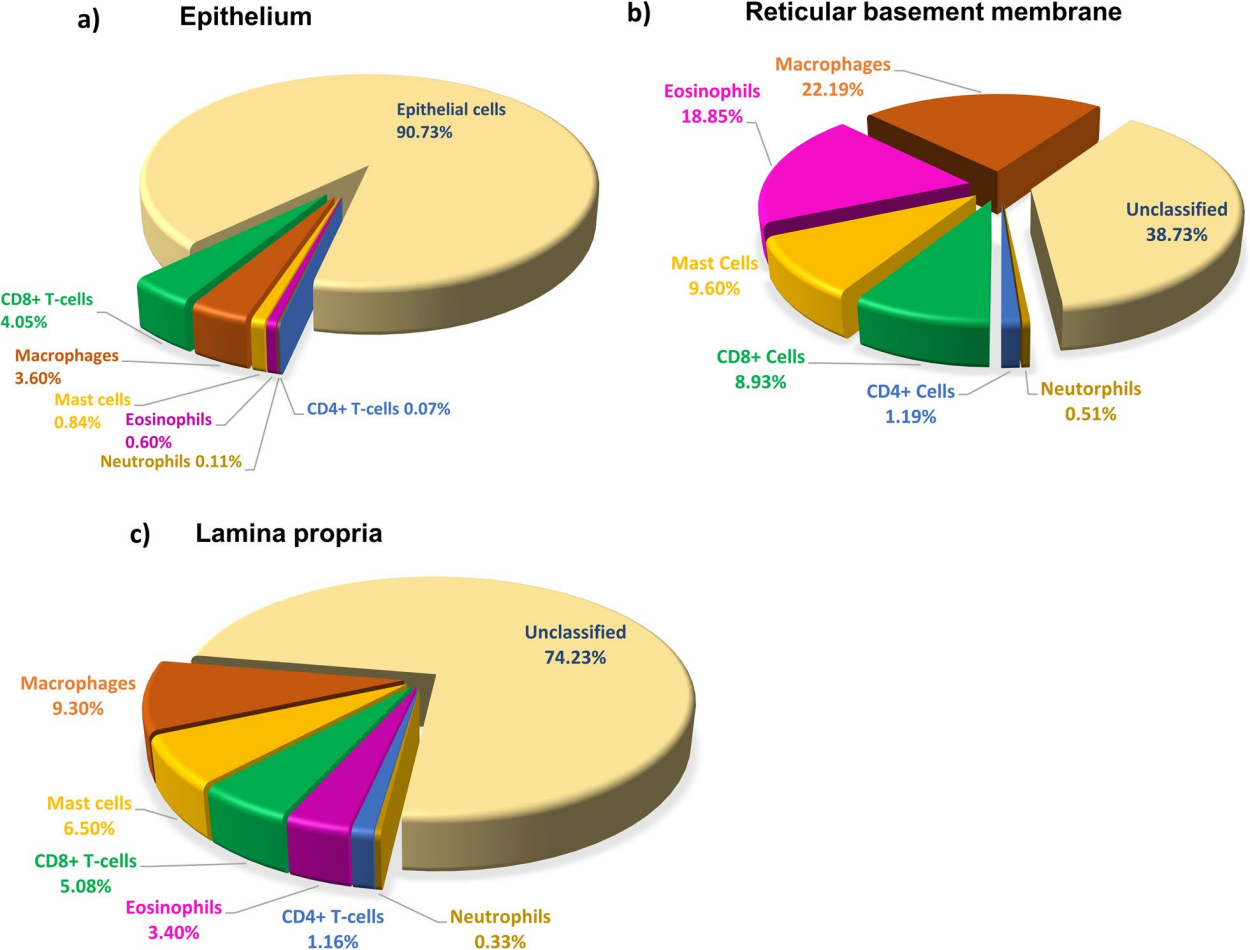
Data showing the percent contribution of inflammatory cells in ACO epithelium (**a**), reticular basement membrane (RBM) (**b**), and LP (**c**)

**Fig. 9:**
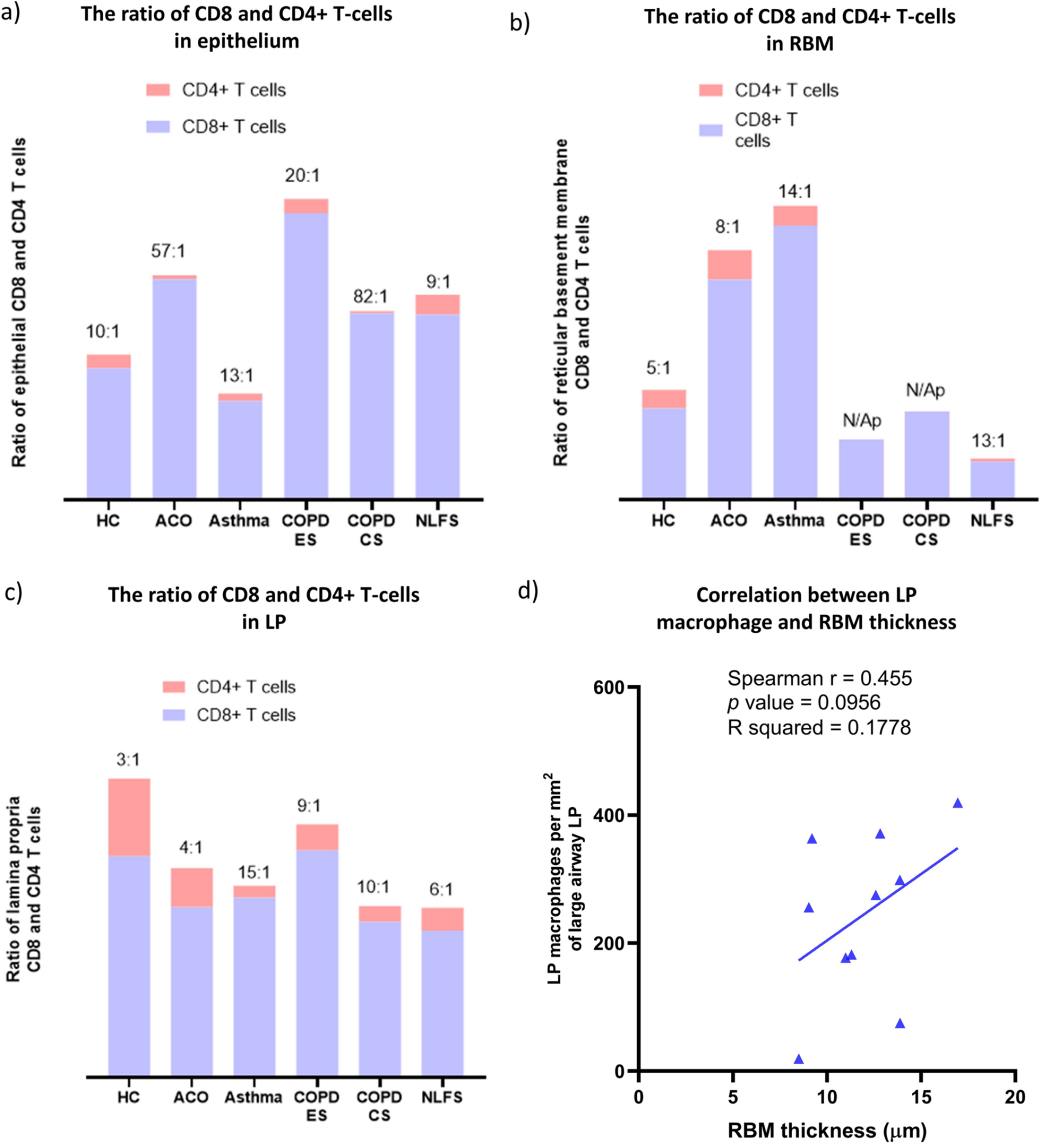
Column plots showing the ratio of CD8+ T-cells and CD4+ T-cells in large airway epithelium (**a**), reticular basement membrane (RBM) (**b**), and lamina propria (LP) (**c**) of healthy control (HC), asthma COPD overlap (ACO), asthma, chronic obstructive pulmonary disease (COPD) ex –smokers (ES) and current smokers (CS), and normal lung function smokers (NLFS). Data showing the nonparametric (Spearman) correlation between ACO LP macrophage and RBM thickness (**d**)

**Fig. 10:**
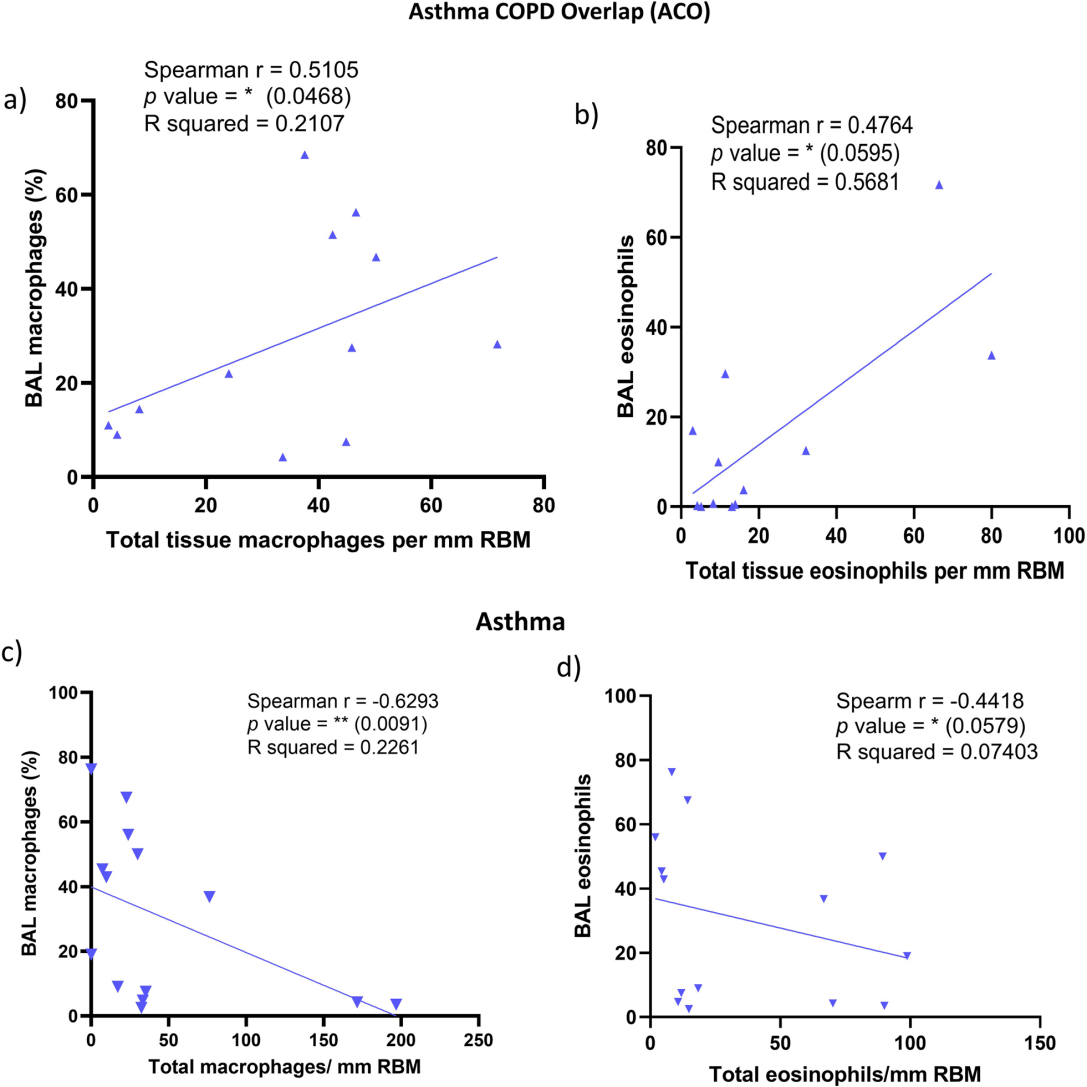
Data showing spearman correlation between bronchoalveolar lavage (BAL) and total tissue macrophage (**a**) and eosinophils (**b**) in ACO. Data showing Spearman correlation between bronchoalveolar lavage (BAL) and total tissue macrophage (**c**) and eosinophils (**d**) in asthma

We checked the contribution of individual inflammatory cell types in the epithelium, RBM, and LP of ACO, and we noted that the macrophages contributed 3.6% in the epithelium, 22.19% in RBM, and 9.3% of total LP cells (Fig. 8a–c). Furthermore, CD8+ T-cells and CD4+ T-cells ratio suggested an accumulation of CD8 + T-cells over CD4 + T-cells in epithelial, RBM, and the LP region of ACO (Fig. 9a–c). When we evaluated HC, the CD8 + /CD4 + ratio tended to be higher in both epithelium and RBM of ACO; however, the ratio tended to be lower in ACO LP than HC. Since we noticed distinguishable differences in macrophage cell numbers, we further examined the correlation (Fig. 9d) between the LP macrophage number and the RBM thickness we previously measured in ACO patients [13]. The result suggested a positive correlation between the LP macrophage number and the RBM thickness (Spearman r 0.455, *P* 0.0956). Furthermore, the results of correlation analysis between macrophages and FEV_1_/FVC suggested a negative correlation between macrophages in RBM (Spearman r -0.32, *P* 0.158) and LP (Spearman r -0.210, *P* 0.257); however, the correlation was not statistically significant in both cases. Our correlation analysis of BAL and total tissue macrophages and eosinophils suggested interesting and contrasting results in patients with ACO (Fig. 10a, b) and asthma (Fig. 10c, d). In ACO, we noted a strong positive and significant correlation between bronchoalveolar lavage (alveolar) and total tissue macrophages (Spearman r 0.5105, *P* 0.0468) and eosinophil (Spearman r 0.4764, *P* 0.0595), whereas, in the patients with asthma, these correlation analyses resulted in a strong negative and significant *P* values (for macrophages Spearman r -0.6293, *P* 0.0091; eosinophils Spearman r -0.4418, *P* 0.0579).

### Effects of inhaled corticosteroids (ICS) on inflammatory cells in the large airways of patients with asthma and asthma-COPD overlap (ACO)

**Fig. 11:**
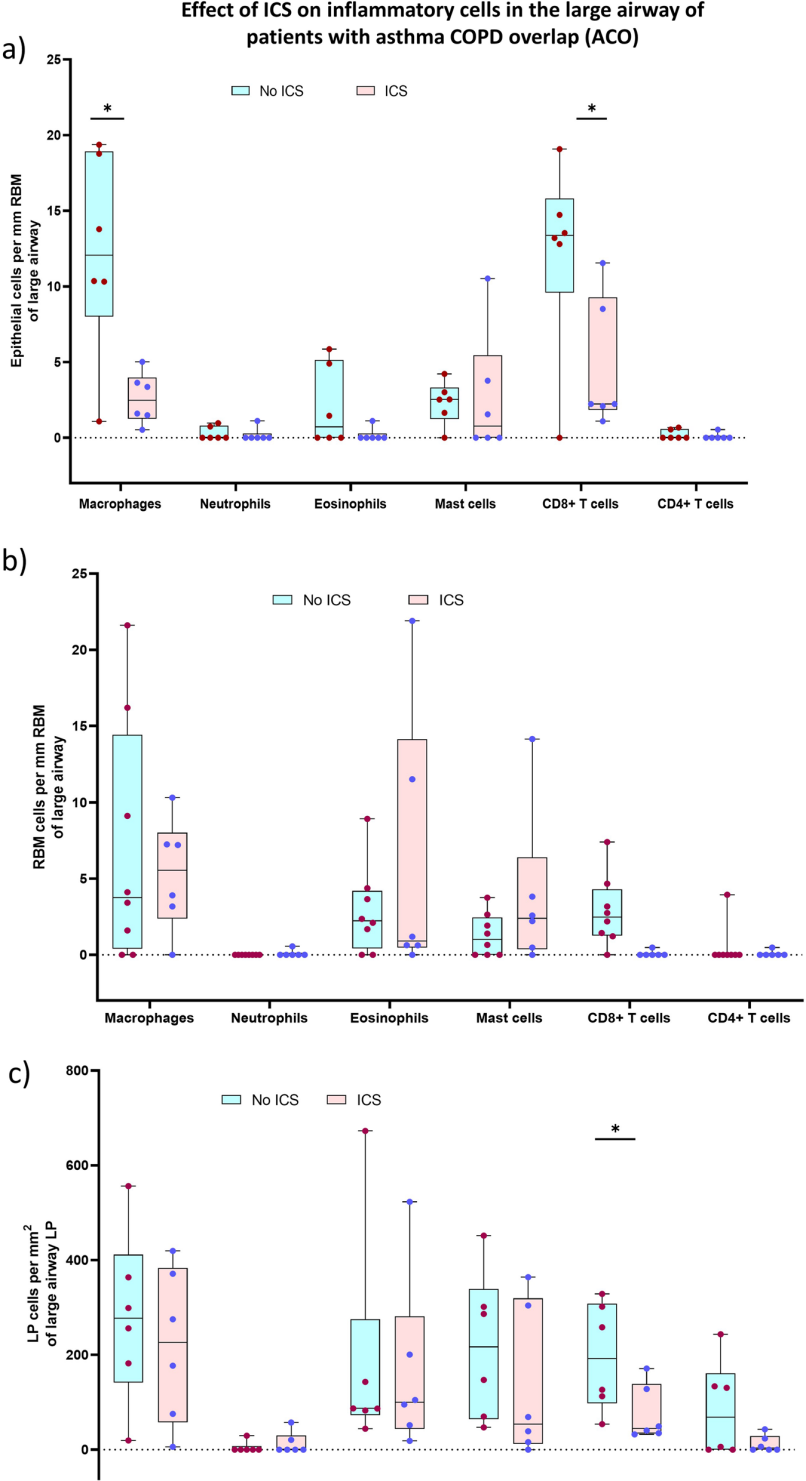
Box plots showing the inflammatory cells in the epithelium (**a**), reticular basement membrane (RBM) (**b**), and lamina propria (LP) (**c**) of ACO with or without ICS treatment. Mann-Whitney test was performed for each inflammatory cell type between the patients with and without ICS treatment, and plots were shown in one pane. *P*-value representation * <0.05

**Fig. 12:**
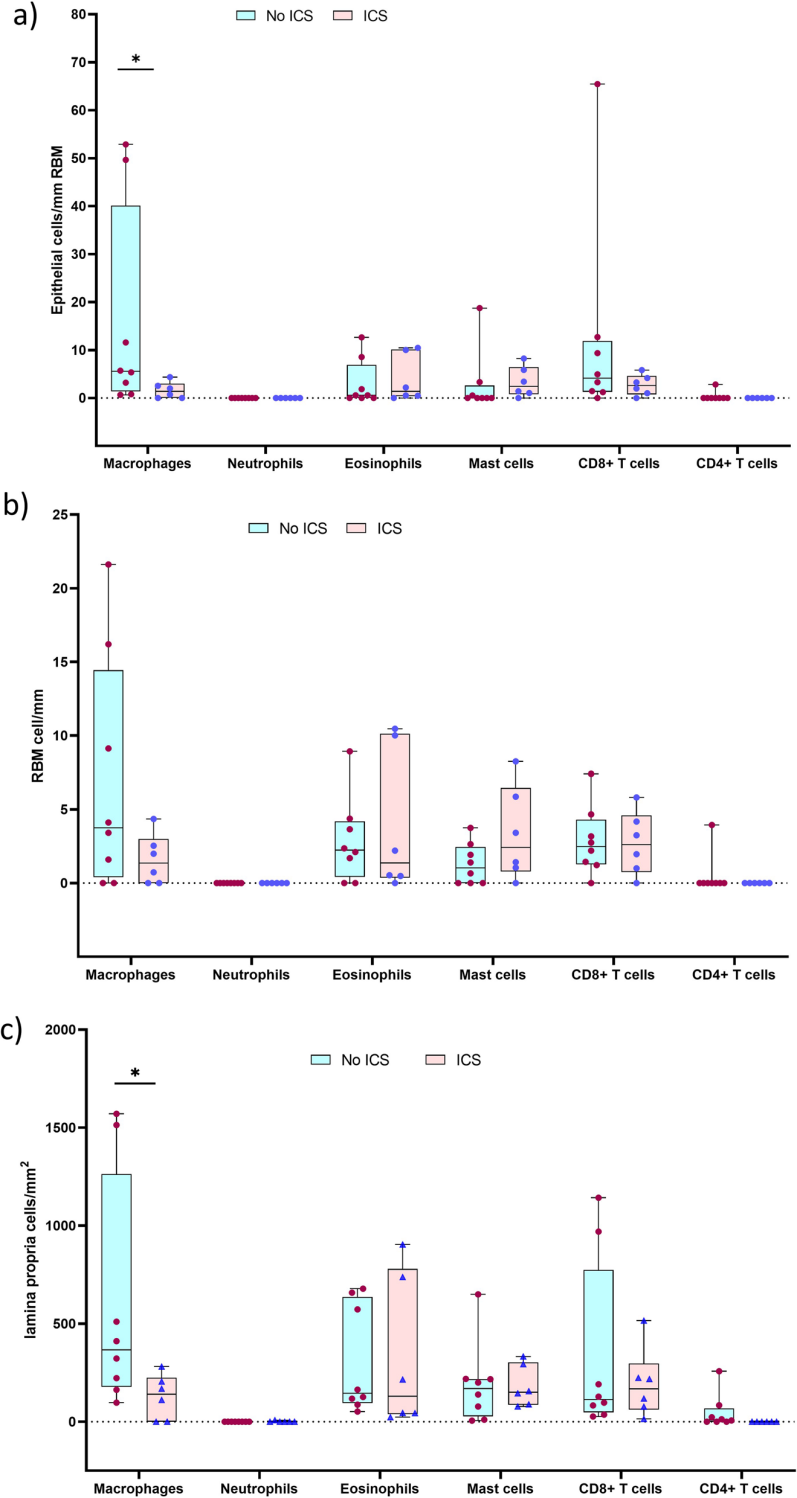
Box plots showing the inflammatory cells in the epithelium (**a**), reticular basement membrane (RBM) (**b**), and lamina propria (LP) (**c**) of asthma with or without ICS treatment. Mann–Whitney test was performed for each inflammatory cell type between the patients with and without ICS treatment and plots were shown in one pane. *P*-value representation * <0.05

**Fig. 13:**
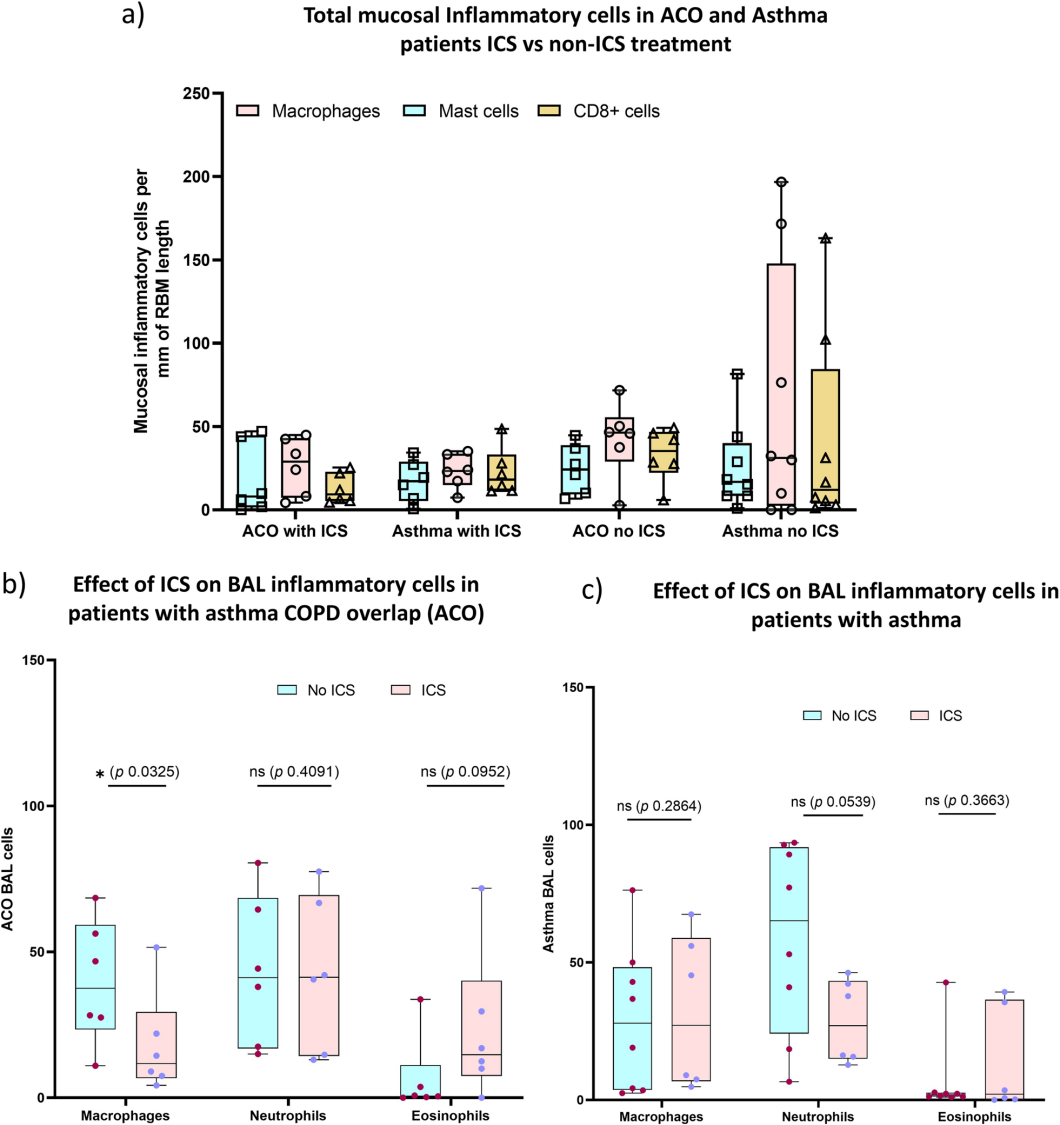
Box plots showing the changes of total mucosal macrophages, mast cells, and CD + T-cells among patents with asthma-COPD overlap (ACO) and asthma in ICS non-treated and ICS-treated patients (**a**). Box plots showing the BAL inflammatory cells of ACO (**b**) and asthma (**c**) with or without ICS treatment. Mann–Whitney test was performed for each inflammatory cell type between the patients with and without ICS treatment and plots were shown in one pane

Since our study included airway tissues from ICS-treated patients with ACO and asthma, we further dichotomized the inflammatory cells evaluated in all three regions of tissue and BAL based on the presence or absence of ICS treatment. Our results suggested low (*P* < 0.05) numbers of epithelial macrophages and CD8 + T-cells in the epithelium and LP of ICS-treated ACO patients (Fig. 11a–c). Similarly, a low number of macrophage was noted in the epithelium and LP of ICS-treated asthmatics (Fig. 12a–c). Based on these results we further checked differences of total mucosal tissue macrophages, mast cells, and CD8 + T-cells (per RBM length) between ACO and asthma groups without ICS and with ICS-treated patients (Fig. 13a). The results indicated no significant difference between these two groups in both with and without ICS-treated patients. However, it appears that the number of tissue macrophages, mast cells, and CD8 + T cells in the non-ICS were variable, with a more extensive spread in asthma than ACO, though we find that the median values do not differ significantly. In addition, the number of BAL macrophages was significantly low in ACO patients treated with ICS than in untreated patients (Fig. 13b). In contrast BAL macrophage numbers were similar between ICS-treated and untreated patients with asthma (Fig. 13c).

## Discussion

Here we provided for the first time a comprehensive assessment of the inflammatory cell profiles in large airway biopsy tissues from patients with ACO and compared them with well-characterised asthma, COPD, NLFS, and HC. Our earlier research reported variable total cellularity in the RBM and LP area of ACO compared to HC. There is growing evidence that inflammatory cells play an important role in airway disease pathogenesis [1, 7]. Important inflammatory cells in ACO airway tissues were previously qualitatively assessed [12]. In this study, we performed meticulous assessments of the critical inflammatory cells such as macrophages, eosinophils, neutrophils, mast cells, and T-cells. Most prominently we identified significant elevation of tissue macrophage numbers in the airway wall epithelium, RBM, and LP of patients with ACO compared to similar regions of our HC, patients with asthma and COPD. Interestingly, we find fluctuating number of eosinophils, neutrophils, mast cells and CD8 + T-cells in the ACO, asthma, and COPD patients, airways compared to HC (Table 2). Given the paucity of original research involving airway tissue of ACO, here we outlay our discussion of how our study results fit into asthma or COPD disease pathology.

**Table 2:** Inflammatory cell count among HC, ACO, asthma, COPD-ES, COPD-CS, and NLFS

	HC	ACO	Asthma	COPD-ES	COPD-CS	NLFS
Macrophage						
Epithelium	0 9 (0.5–9.3)	4.3 (0.5–19.4)	1.2 (0–11.6)	0.9 (0–3.2)	1.5 (0–11.7)	3.2 (0–11.9)
RBM	0.5 (0–2.7)	4.3 (0–13.4)	1.8 (0–9.1)	0.2 (0.-2.8)	0 (0–2.3)	0.1 (0–4.9)
LP	192.1 (33.0–429.7	265.6 (6.0–556.2)	186.8 (0–510.7)	170.5 (31.6–436.2)	124.6 (18.9–307.1)	206.9 (13.4–429.4)
Neutrophils						
Epithelium	0.5 (0–10.3)	0 (0–1.1)	0	0.4 (0–2.1)	0 (0–7.6)	0.5 (0–5.7)
RBM	3.0 (0–13.3)	0 (0–1.0)	0	0.9 (0–7.0)	0.6 (0–10.3)	2.5 (0–9.6)
LP	128.8 (0–637.7)	0 (0–57.5)	0 (0–7.9)	73.24 (0–260.5)	26.25 (0–403.7)	95.84 (19.8–184.7)
Eosinophils						
Epithelium	1.4 (0–3.5)	0.3 (0–5.9)	0.6 (0–12.9)	0.3 (0–1.9)	0.5 (0–13.3)	0.8 (0–4.3)
RBM	1.3 (0–6.1)	0.9 (0–21.9)	2.2 (0–10.5)	0.6 (0–3.7)	1.0 (0–16.1)	1.2 (0–8.5)
LP	185.8 (24.6–394.6)	87.01 (18.4–200.5)	144.7 (22.6–903.8)	52.73 (23.8–131.4)	70.40 (0–301.6)	110.7 (12.4–405.7)
Mast cells						
Epithelium	4.3 (0–20.3)	1.7 (0–4.2)	0 (0–11.9)	4.7 (0–23.2)	5.7 (0–14.0)	6.5 (0–20.2)
RBM	3.7 (0.5–5.4)	2.2 (0–5.5)	1.4 (0–8.3)	4.6 (0.8–15.9)	6.4 (0–11.3)	8.5 (0.8–14.9)
LP	305.9 (99.6–549.6)	108.5 (0–451-8)	150.2 (5.6–649.6)	267.5 (48.6–551.6)	256.9 (0–374.5)	265.9(140.4–481.0)
CD8 + T-cells						
Epithelium	3.7 (0–13.6)	10.0 (0–19.1)	3.3 (0–12.7)	5.0 (0–38.2)	5.4 (0–23.0)	6.7 (0–21.8)
RBM	0 (0- 4.0)	1.7 (0–5.8)	2.5 (0–7.4)	0.3 (0–1.9	0.6 (0–2.1)	0 (0–1.1)
LP	116.6 (24.1–532.2	119.5 (32.3–328.8)	107.4 (13.9–515.6)	180.0 (38.0–417.0)	136.2 (14.7–183.0)	93.62 (4.9–319.3)
CD4 + T-cells						
Epithelium	0 (0–1.7)	0 (0–0.7)	0 (0–4.0)	0 (0–3.2)	0 (0–1.0)	0 (0–4.2)
RBM	0 (0–1.2)	0 (0–1.9)	0 (0–2.8)	0	0	0 (0–0.4)
LP	12.5 (0–217.2)	6.0 (0–133.7)	0 (0–83.4)	0.8 (0–118)	0 (0–98.9)	6.3 (0–84)

Data presented as median (minimum – maximum)

*ACO* asthma COPD overlap, *COPD* chronic obstructive pulmonary disease, *COPD-CS* COPD current smokers, *COPD-ES.* COPD ex-smokers, *RBM* reticular basement membrane, *LP* lamina propria, *NLFS* normal lung function smoker

Macrophages are workhorses, playing an important role in phagocytosis and to some extent, they play a paradoxical functional role in both pro and anti-inflammatory processes [14]. Recently, Moghbelia et al. [15] reported that adrenergic β-agonist exposure preferentially suppresses the BAL macrophage cAMP gene module in asthma and ACO suggesting suppression of lung macrophages beneficial for ACO patients; a similar effect could also be expected in tissue macrophages. However, studies with induced sputum showed a generally lower level of macrophage and a higher level of neutrophil gelatinase-associated lipocalin (NGAL), a neutrophil derived inflammatory molecule, in patients with ACO compared to asthma, COPD, and healthy [16–18]. Although speculative, the possible egress of macrophages into the airway lumen from airway tissue that entered from blood circulation could not be ruled out [19]. In contrast, the increase in serum IL-18 was found to be associated with a decrease in FEV1 and TNF-α with FEV_1_/FVC (%) in ACO patients suggesting a possible role of the macrophage in systemic inflammation and pathogenesis of ACO [20, 21]. Further mechanistic research is required to confirm the role and function of macrophages in ACO. In COPD patients, macrophages are prominent and increased in number in the bronchial submucosa. Their numbers increased with the increasing disease severity [22–24]. Our previous findings suggested an abnormal macrophage shift in both mucosal and luminal areas of the airway in COPD patients, which was strongly associated with cytokine balance [9]. However, the literature also indicated variability of macrophages in the subepithelial region [22, 25]. The findings of mucosal macrophages in asthma patients are conflicting [26–30]. A few studies have reported higher macrophages than eosinophils, the later inflammatory cells are generally considered dominant in eosinophilic asthma patients. Others have reported lower tissue macrophages or similar number of macrophages in asthma patients compared to normal healthy controls. Macrophages are also responsible for modulating extracellular matrix proteins which were proven to have implications on RBM thickness [1, 7, 31]. Indeed, we noted a positive near significant correlation between macrophage number in ACO LP and RBM thickness that was previously reported to be thicker in ACO [13].

Mast cells and eosinophils are vital effector cells in asthma; however, neither of these two cells has been ascribed a pronounced role in COPD [1, 32]. A low tryptase-positive mast cells in ACO in our study may be explained by the fact that most of ACO patients in our study are nonatopic. However, upregulation of mast cell activation pathways could also be hypothesized in ACO considering the high serum IgE levels in ACO compared to COPD and healthy [33]. We currently cannot explain the low mast cell number in asthmatic mucosa, even though, most of our asthma patients were atopic. As indicated in the results section, our low mast cell count in ACO or asthma was not significantly influenced by the ICS treatment. Our findings on low mast cells in COPD LP agree with findings by Gosman et al. [34], who also reported a low mast cell density in COPD LP of the central airway compared with control subjects; however, this contradicts previous results by Soltani et al. [35].

Our findings of low ACO tissue eosinophilia were similar to the mild to moderate tissue eosinophilia in half of the ACO patient, reported in a study [12] which did not have a comparison against HC. Researchers also reported the clinical utility of blood eosinophilia with FeNO for differentiating ACO from COPD or asthma [36, 37]. However, blood eosinophil count was found to not differentiate between asthma, COPD, and ACO in the largest comparative cohort NOVELTY study [38]. Tissue eosinophilia is the hallmark of asthma [1, 6]. In our study, approximately 35% of patients in the asthma group showed a trend of generally higher tissue eosinophil count than the HC suggesting the heterogenic nature of the disease condition (Fig. 4b–d). Interestingly, the majority of the asthma patients showed a lower eosinophil count in BAL among the BAL cells counted. More importantly, eosinophilia in induced sputum has been reported in many studies [16, 39], which could be suggestive of the ingress theory [19] of eosinophils from tissue to airway lumen.

The results BAL and tissue macrophages and eosinophils in the ACO and asthma groups showed a contrasting trend and encouraging. These results seem to support the hypothesis that both epithelial and subepithelial inflammation play an active role in ACO whereas an active mucosal inflammation in asthma. Further, research is required to understand the mechanism behind this observation.

Neutrophils and their products are prominent inflammatory mediators for ACO, COPD, and a subset of asthma [1]; but, we found very low mucosal neutrophil numbers in ACO compared to HC which is consistent across asthma or COPD although our BAL ACO neutrophils were noticeable. Studies also indicated a higher neutrophil number or NGAL in patients with ACO [17, 40]. Also, some of the previous studies failed to show a difference between patients with asthma or COPD and control with a trend of high neutrophil numbers in the control group in one of the biopsy studies [22, 26, 29, 41, 42].

Both CD8+ T-cells and CD4+ T-cellsave direct effector activity including performing cytotoxic functions [43]. Generally, activated (eg, due to antigenic stimulus) memory T-cells linger in the lung and contribute to the host defence [44]. The epithelium, being the primary protective layer of the lung, is constantly exposed to the external environment and becomes the major entry port of many pathogens during the respiratory process. Although the luminal macrophage removes pathogens through their phagocytic mechanisms, indeed, we noted a high macrophage in BAL fluid, the pathogen that escapes the primary phagocytic process, requires innate CD4 + or CD8 + T-cells to control the infection. Our observation of insignificantly higher CD8 + T-cell numbers in the epithelium and RBM of ACO than HC could reflect the susceptibility of the ACO airway towards viral infection. Furthermore, our observations on CD8+ T-cells to CD4+ T-cells ratios suggest that ACO airway is immunologically skewed towards CD8 + T-cells with the maximum skewness in the epithelial region suggesting a role of intraepithelial lymphocytes in the long-term inflammatory response [45].

Although, most studies [29, 34, 42] reported the low tissue neutrophils, mast cells, and eosinophils in their control groups, these cells were higher in our HC group. Our findings of these cells are supported by the findings reported by N Carroll et al. [46]. Healthy control subjects in our study were volunteers for research purposes and were recruited and assessed by experienced respiratory physicians. They had not reported previous history of respiratory illness or smoking history. These subjects are recruited based on lung physiological parameters assessed by well-established spirometry methods in a hospital setting [13]. We have previously published [8, 47], that airway wall in smokers and COPD patients is hypocellular due to decrease in key inflammatory cell populations. Normal subjects are not actually showing high levels of immune cells but maintaining a normal immune profile.

The current research is limited to the more highly cited ‘classical’ inflammatory cells in asthma or COPD. It may be argued that ICS treatment had a confounding effect on data rather than the disease; however, we believe that without the ICS treatment, the inflammatory cell numbers could be even higher than that we observed in this study, which was supported by our results of ACO, and asthma non-ICS treated comparison. Furthermore, we found a minimum or no difference with or without ICS treatment on eosinophil in patients with asthma although ICS treatment is a standard treatment in this patient population; however, possibility of resistant to ICS treatment could be a contributing factor [48]. The study also has the limitation of Type II error, as we have seen the absence of statistically significant differences among the groups; however, we have also observed significant differences among the groups representing robust distinctions. We acknowledge that bronchodilator reversibility data are limited to only ACO patients.

## Conclusions

Overall, we believe that our findings do demonstrate that, in ACO patients the tissue inflammatory cell profile differed based on cell type and the location in the mucosa than that of the contributing diseases. We believe that the current research findings would be helpful to the clinician for their informed decision-making process. Our findings also strongly suggest that in the airway wall of ACO patient’s macrophage are crucial, possibly contributing to the structural changes. Targeting them using conventional and novel treatments could improve the clinical management of this patient group. However, further studies based on large populations are required for a more definitive answer considering the heterogenicity of these airway diseases.

## Data Availability

The datasets used and analyzed during the current study are available from the corresponding author upon reasonable request.

## Abbreviations

ACO: Asthma-COPD overlap
BAL: Bronchoalveolar lavage
COPD: Chronic obstructive pulmonary disease
CS: Current smokers
EBB: Endobronchial biopsy
ES: Ex-smokers
HC: Healthy control
ICS: Inhaled corticosteroids
RBM: Reticular basement membrane
LP: Lamina propria
NGAL: Gelatinase-associated lipocalin
NOVELTY: The NOVEL observational Longitudinal Study
NLFS: Normal lung function smokers

## References

[1] Dey S, Eapen MS, Chia C, Gaikwad AV, Wark PAB, Sohal SS (2021). Pathogenesis, clinical features of asthma COPD overlap (ACO), and therapeutic modalities. Am J Physiol Lung Cell Mol Physiol..

[2] GINA. Global initiative for asthma. 2022 https://ginasthma.org/gina-reports/. Accessed 2023

[3] Global initiative for chronic obstructive pulmonary disease. https://goldcopd.org/2022-gold-reports-2/. Accessed 17 Feb 2023.

[4] Kim J, Kim YS, Kim K, Oh YM, Yoo KH, Rhee CK (2017). Socioeconomic impact of asthma, chronic obstructive pulmonary disease and asthma-COPD overlap syndrome. J Thorac Dis.

[5] Jeffery PK, Laitinen A, Venge P (2000). Biopsy markers of airway inflammation and remodelling. Respir Med.

[6] Sutherland ER, Martin RJ (2003). Airway inflammation in chronic obstructive pulmonary disease: comparisons with asthma. J Allergy Clin Immunol.

[7] Eapen M, Myers S, Walters E, Sohal S (2017). Airway inflammation in chronic obstructive pulmonary disease (COPD): a true paradox. Expert Rev Respir Med.

[8] Eapen MS, McAlinden K, Tan D, Weston S, Ward C, Muller HK (2017). Profiling cellular and inflammatory changes in the airway wall of mild to moderate COPD. Respirology.

[9] Eapen MS, Hansbro PM, McAlinden K, Kim RY, Ward C, Hackett TL (2017). Abnormal M1/M2 macrophage phenotype profiles in the small airway wall and lumen in smokers and chronic obstructive pulmonary disease (COPD). Sci Rep.

[10] Uddin M, Watz H, Malmgren A, Pedersen F (2019). NETopathic inflammation in chronic obstructive pulmonary disease and severe asthma. Front Immunol.

[11] Higham A, Leow-Dyke S, Jackson N, Singh D (2020). Stability of eosinophilic inflammation in COPD bronchial biopsies. Eur Respir J..

[12] Papakonstantinou E, Savic S, Siebeneichler A, Strobel W, Jones PW, Tamm M (2019). A pilot study to test the feasibility of histological characterisation of asthma-COPD overlap. Eur Respir J.

[13] Dey S, Lu W, Weber HC, Young S, Larby J, Chia C (2022). Differential airway remodelling changes were observed in patients with asthma COPD overlap (ACO) compared to asthma and COPD patients alone. Am J Physiol Lung Cell Mol Physiol..

[14] Hou F, Xiao K, Tang L, Xie L (2021). Diversity of macrophages in lung homeostasis and diseases. Front Immunol.

[15] Moghbeli K, Valenzi E, Naramore R, Sembrat JC, Chen K, Rojas MM (2021). β-Agonist exposure preferentially impacts lung macrophage cyclic AMP-related gene expression in asthma and asthma COPD overlap syndrome. Am J Physiol Lung Cell Mol Physiol.

[16] Gao J, Zhou W, Chen B, Lin W, Wu S, Wu F (2017). Sputum cell count: biomarkers in the differentiation of asthma, COPD and asthma-COPD overlap. Int J Chron Obstruct Pulmon Dis.

[17] Iwamoto H, Gao J, Koskela J, Kinnula V, Kobayashi H, Laitinen T (2014). Differences in plasma and sputum biomarkers between COPD and COPD-asthma overlap. Eur Respir J.

[18] Kyogoku Y, Sugiura H, Ichikawa T, Numakura T, Koarai A, Yamada M (2019). Nitrosative stress in patients with asthma-chronic obstructive pulmonary disease overlap. J Allergy Clin Immunol.

[19] Persson C, Uller L (2010). Transepithelial exit of leucocytes: inflicting, reflecting or resolving airway inflammation?. Thorax.

[20] Kubysheva N, Boldina M, Eliseeva T, Soodaeva S, Klimanov I, Khaletskaya A (2020). Relationship of serum levels of IL-17, IL-18, TNF-α, and lung function parameters in patients with COPD, Asthma-COPD overlap, and bronchial asthma. Mediators Inflamm.

[21] Fricker M, Gibson PG (2017). Macrophage dysfunction in the pathogenesis and treatment of asthma. Eur Respir J.

[22] Saetta M, Di Stefano A, Maestrelli P, Ferraresso A, Drigo R, Potena A (1993). Activated T-lymphocytes and macrophages in bronchial mucosa of subjects with chronic bronchitis. Am Rev Respir Dis.

[23] van den Berge M, Vonk JM, Gosman M, Lapperre TS, Snoeck-Stroband JB, Sterk PJ (2012). Clinical and inflammatory determinants of bronchial hyperresponsiveness in COPD. Eur Respir J.

[24] Hogg JC, Chu F, Utokaparch S, Woods R, Elliott WM, Buzatu L (2004). The nature of small-airway obstruction in chronic obstructive pulmonary disease. N Engl J Med.

[25] Rutgers SR, Postma DS, ten Hacken NH, Kauffman HF, van Der Mark TW, Koëter GH (2000). Ongoing airway inflammation in patients with COPD who do not currently smoke. Thorax.

[26] Poston RN, Chanez P, Lacoste JY, Litchfield T, Lee TH, Bousquet J (1992). Immunohistochemical characterization of the cellular infiltration in asthmatic bronchi. Am Rev Respir Dis.

[27] Lane SJ, Sousa AR, Lee TH (1994). The role of the macrophage in asthma. Allergy.

[28] Vignola AM, Chanez P, Chiappara G, Siena L, Merendino A, Reina C (1999). Evaluation of apoptosis of eosinophils, macrophages, and T lymphocytes in mucosal biopsy specimens of patients with asthma and chronic bronchitis. J Allergy Clin Immunol.

[29] Bradley BL, Azzawi M, Jacobson M, Assoufi B, Collins JV, Irani AM (1991). Eosinophils, T-lymphocytes, mast cells, neutrophils, and macrophages in bronchial biopsy specimens from atopic subjects with asthma: comparison with biopsy specimens from atopic subjects without asthma and normal control subjects and relationship to bronchial hyperresponsiveness. J Allergy Clin Immunol.

[30] Chanez P, Vago P, Demoly P, Cornillac L, Godard P, Bureau JP (1993). Airway macrophages from patients with asthma do not proliferate. J Allergy Clin Immunol.

[31] Wight TN, Frevert CW, Debley JS, Reeves SR, Parks WC, Ziegler SF (2017). Interplay of extracellular matrix and leukocytes in lung inflammation. Cell Immunol.

[32] Virk H, Arthur G, Bradding P (2016). Mast cells and their activation in lung disease. Transl Res.

[33] Kalinina EP, Denisenko YK, Vitkina TI, Lobanova EG, Novgorodtseva TP, Antonyuk MV (2016). The mechanisms of the regulation of immune response in patients with comorbidity of chronic obstructive pulmonary disease and asthma. Can Respir J.

[34] Gosman MM, Postma DS, Vonk JM, Rutgers B, Lodewijk M, Smith M (2008). Association of mast cells with lung function in chronic obstructive pulmonary disease. Respir Res.

[35] Soltani A, Ewe YP, Lim ZS, Sohal SS, Reid D, Weston S (2012). Mast cells in COPD airways: relationship to bronchodilator responsiveness and angiogenesis. Eur Respir J.

[36] Takayama Y, Ohnishi H, Ogasawara F, Oyama K, Kubota T, Yokoyama A (2018). Clinical utility of fractional exhaled nitric oxide and blood eosinophils counts in the diagnosis of asthma-COPD overlap. Int J COPD.

[37] Perez-de-Llano L, Cosio BG (2017). Asthma-COPD overlap is not a homogeneous disorder: further supporting data. Respir Res.

[38] Reddel HK, Vestbo J, Agustí A, Anderson GP, Bansal AT, Beasley R (2021). Heterogeneity within and between physician-diagnosed asthma and/or COPD: NOVELTY cohort. Eur Respir J..

[39] Green RH, Brightling CE, McKenna S, Hargadon B, Parker D, Bradding P (2002). Asthma exacerbations and sputum eosinophil counts: a randomised controlled trial. Lancet.

[40] Wang J, Lv H, Luo Z, Mou S, Liu J, Liu C (2018). Plasma YKL-40 and NGAL are useful in distinguishing ACO from asthma and COPD. Respir Res.

[41] O'Shaughnessy TC, Ansari TW, Barnes NC, Jeffery PK (1997). Inflammation in bronchial biopsies of subjects with chronic bronchitis: inverse relationship of CD8+ T lymphocytes with FEV1. Am J Respir Crit Care Med.

[42] Lacoste JY, Bousquet J, Chanez P, Van Vyve T, Simony-Lafontaine J, Lequeu N (1993). Eosinophilic and neutrophilic inflammation in asthma, chronic bronchitis, and chronic obstructive pulmonary disease. J Allergy Clin Immunol.

[43] Seder RA, Ahmed R (2003). Similarities and differences in CD4+ and CD8+ effector and memory T cell generation. Nat Immunol.

[44] Woodland DL, Scott I (2005). T cell memory in the lung airways. Proc Am Thorac Soc.

[45] Löfdahl MJ, Roos-Engstrand E, Pourazar J, Bucht A, Dahlen B, Elmberger G (2008). Increased intraepithelial T-cells in stable COPD. Respir Med.

[46] Carroll N, Lehmann E, Barret J, Morton A, Cooke C, James A (1996). Variability of airway structure and inflammation in normal subjects and in cases of nonfatal and fatal asthma. Pathol Res Pract.

[47] Sohal SS, Reid D, Soltani A, Weston S, Muller HK, Wood-Baker R (2013). Changes in airway histone deacetylase2 in smokers and COPD with inhaled corticosteroids: a randomized controlled trial. PLoS ONE.

[48] Barnes PJ (2013). Corticosteroid resistance in patients with asthma and chronic obstructive pulmonary disease. J Allergy Clin Immunol.

